# Assessment of Sarcopenia Related Quality of Life Using SarQoL® Questionnaire in Patients With Liver Cirrhosis

**DOI:** 10.3389/fnut.2022.774044

**Published:** 2022-02-25

**Authors:** Speranta Iacob, Victor Mina, Matei Mandea, Razvan Iacob, Roxana Vadan, Voichita Boar, Georgeta Ionescu, Dan Buzescu, Cristian Gheorghe, Liana Gheorghe

**Affiliations:** ^1^“Carol Davila” University of Medicine and Pharmacy, Bucharest, Romania; ^2^Center for Digestive Diseases and Liver Transplant, Fundeni Clinical Institute, Bucharest, Romania; ^3^Dr Carol Davila Central Military Emergency Hospital, Bucharest, Romania; ^4^Colentina Clinical Hospital, Bucharest, Romania

**Keywords:** quality of life, sarcopenia, cirrhosis, liver, frailty

## Abstract

**Introduction:**

Sarcopenia, malnutrition, physical deconditioning, and frailty contribute to a significantly altered quality of life (QoL) in patients with cirrhosis and sarcopenia.

**Aim:**

To investigate the sarcopenia-linked alterations of QoL by SarQoL® questionnaire in patients with end-stage liver disease.

**Methods:**

Consecutive patients with liver cirrhosis, admitted to our department between May and August 2021, completed the SarQoL® questionnaire by themselves. They were evaluated for sarcopenia according to the 2019 European Working Group on Sarcopenia in Older People (EWGSOP) definition [hand grip cut-offs and skeletal muscle index (SMI) calculation at CT scan].

**Results:**

A total of 71 patients with liver cirrhosis were included in the study, with a median age of 54 years. Sarcopenia was present in 31.2% of patients with Child-Pugh class A, in 58.3% with class B, and in 93.5% with class C. The SarQoL® score was statistically significant and lower in Child-Pugh class C vs. class B and class A (70.2 vs. 66.5 vs. 52.5 points, *p* = 0.0002). The SarQoL® score was evaluated according to different complications of cirrhosis, with statistically significant lower scores in patients with sarcopenia (*p* < 0.0001), in patients with ascites requiring paracentesis (*p* = 0.0006), and in patients with hepatic encephalopathy (*p* < 0.0001). A cut-off level of 75.9 points for SarQoL® score can accurately detect sarcopenia in patients with end-stage liver disease [area under the receiver operating characteristic (AUROC) curve of.823, SE of 92.1%, SP of 45.5%, positive predictive value (PPV) and negative predictive value (NPV) of 66 and 83.3%, respectively, correctly classified 73.2% of cirrhotic patients with sarcopenia].

**Conclusions:**

The use of SarQoL® questionnaire in cirrhotic patients can, at the same time, evaluate the quality of life and identify subjects with sarcopenia and altered QoL.

## Introduction

The prevalence of sarcopenia in cirrhosis ranges from 30–70%, depending on the diagnostic tools utilised and the severity of the underlying liver disease (1). Sarcopenia is defined as a progressive and generalised skeletal muscle disorder associated with an increased likelihood of adverse outcomes including falls, fractures, disability, and mortality (2). It is associated with higher rates of complications of cirrhosis (especially hepatic encephalopathy and infections), hospital admissions, and premature mortality (3).

However, muscle strength is better recognised as being associated with adverse outcomes compared to low muscle mass (4).

Sarcopenia proved to risk-stratify patients with cirrhosis independent of the Child-Pugh score and the model for end-stage liver disease (MELD) score and thus may serve as an independent prognostic marker (5). It is associated with poorer clinical outcomes after liver transplantation (LT) (e.g., higher incidence of postoperative sepsis, neurological complications, ventilator support, rejection, length of ICU, and hospital stay and mortality) in addition to a reduced quality of life (QoL) and lack of functional independence (6).

Sarcopenia is the central and dominant component of frailty that influences pre-and post-liver transplant survival. The inclusion of functional measures in validated frailty metrics suggests that the influence of sarcopenia may be modified by factors related to muscle function rather than purely muscle mass according to Lai et al. (7).

There is a strong interplay between sarcopenia, malnutrition, physical deconditioning, and frailty in cirrhosis. All these contribute to a significantly altered QoL in patients with cirrhosis and sarcopenia (8). The health-related QoL of patients with end-stage liver disease is significantly impaired when compared to healthy controls or patients with chronic liver disease as was recently proven in a systematic review (9).

In comparison to other end-stage organ failure or terminal illnesses, the end-stage liver disease disproportionately affects younger age groups, and the years of life lost is estimated to be around 20 years (10, 11). Although these patients are vulnerable and at high risk of death, little attention has been paid to describing their symptom prevalence and health-related QoL.

This indicates the importance of preventive and interventional management strategies for managing patients with liver diseases with compensated or decompensated liver diseases by a multidisciplinary team.

The consequences of sarcopenia on QoL, disability, and mortality are important, and it is recommended that physicians should consider screening for sarcopenia in different settings (12). Only specific domains of QoL are impacted by sarcopenia. Therefore, generic tools may not be able to detect the subtle effects of sarcopenia on QoL. Usually, generic health-related QoL tools (the Medical Outcome Study Questionnaire 36-Item Short Form Survey) were more frequently administered than disease-specific health-related QoL tools (the Chronic Liver Disease Questionnaire) in cirrhosis. Given the diversity of symptoms and significantly impaired health-related QoL, a multidisciplinary approach and timely intervention are crucial (9). In 2015, Beaudart and colleagues (13) reported the development of the first disease-specific self-administrated sarcopenia-related QoL questionnaire, the SarQoL® questionnaire. The SarQoL® is a valid, consistent, and reliable tool that can be used for clinical and research purposes as it proved to be able to discriminate sarcopenic of non-sarcopenic subjects with regard to their QoL, regardless of the definition used for diagnosis as long as the definition includes an assessment of both muscle mass and muscle function.

The aim of our study was to investigate the sarcopenia-linked alterations of QoL by SarQoL® questionnaire in patients with end-stage liver disease.

## Methods

We included consecutive patients with liver cirrhosis admitted to the Hepatology Department of Fundeni Clinical Institute between May and August 2021. Liver cirrhosis was defined based on clinical, biochemical, abdominal ultrasound, and CT scan features. Patients completed the SarQoL® questionnaire by themselves in Romanian language and they were evaluated for sarcopenia according to the 2019 European Working Group on Sarcopenia in Older People (EWGSOP) definition: hand grip cut-offs <26 kg for men and <18 kg for women (4) and skeletal muscle index (SMI) calculation at CT scan. Patients were evaluated for the severity of liver disease (Child-Pugh and MELD-Na scores) and associated complications. An SMI with <39 cm^2^/m^2^ in women and with <50 cm^2^/m^2^ in men were used to define sarcopenia in cirrhosis. The Liver Frailty Index (LFI) was calculated for all patients. The LFI has established the cut-points to define robust (LFI < 3.2), prefrail (LFI 3.2–4.3), and frail (LFI ≥ 4.4), according to the American Association for the Study of Liver Diseases (AASLD) guidelines on malnutrition, sarcopenia, and frailty in cirrhosis (2).

### The SarQoL® Questionnaire

The SarQoL® questionnaire is a patient-reported outcome measure specific to sarcopenia in aged people. The SarQoL® questionnaire consists of 22 questions incorporating 55 items that fall into seven domains of health-related quality of life (HRQoL). These domains are “Physical and Mental Health,” “Locomotion,” “Body Composition,” “Functionality,” “Activities of Daily Living,” “Leisure activities,” and “Fears,” and it takes 10 min to complete. It is available in 16 languages, including Romanian (14). Most questions (19 out of 22) use a Likert scale of frequency or intensity, among which the respondents choose the answer most applicable to them. Each domain is scored from 0 to 100, and an overall score is calculated. We obtained the official scoring algorithm from the developers of the SarQoL® questionnaire (13). The questionnaire has demonstrated its ability to differentiate between sarcopenic and non-sarcopenic subjects (discriminative power) (15).

The authors (13) that developed this questionnaire used the definition of sarcopenia based on the handgrip strength SMI assessed by Dual-energy X-ray absorptiometry (DEXA). However, in patients with cirrhosis, DEXA has major limitations due to its inability to differentiate water from muscle. In order to fulfil all the requirements of sarcopenia evaluation, we included in the definition the calculation of SMI at the CT scan performed routinely in our clinic for all patients with liver cirrhosis.

Our patients completed the questionnaire in their ward, in a quiet atmosphere, alone, after a period of 10 min of rest.

### Statistical Analysis

Statistical analysis was performed using SPSS V20.0 (SAS Institute Inc., Cary, NC, USA) software. Continuous data are expressed as mean ± SD unless otherwise indicated. Categorical data are described as frequencies of the subjects with a specific characteristic. The comparison of categorical parameters was determined by the two-tailed χ^2^-test or Fisher's exact test. Normally distributed continuous parameters were compared with Student's *t*-test, and non-normally distributed continuous parameters were compared with the Mann–Whitney *U* test. Univariate analysis was carried out to identify variables that were significantly different between patients with and without sarcopenia.

The Kruskal-Wallis test was performed for global comparison of quantitative variables.

The diagnostic value of the SarQoL® score for sarcopenia was assessed by calculating the area under the receiver operating characteristic (AUROC) curve. The diagnostic accuracy was calculated using sensitivity (SE), specificity (SP), positive predictive value (PPV), negative predictive value (NPV), and likelihood ratio (LR). The cut-off value for SarQoL® score was chosen to maximise sensitivity in order to use it as a screening tool in patients with liver cirrhosis compensated or decompensated.

## Results

**Figure 1:**
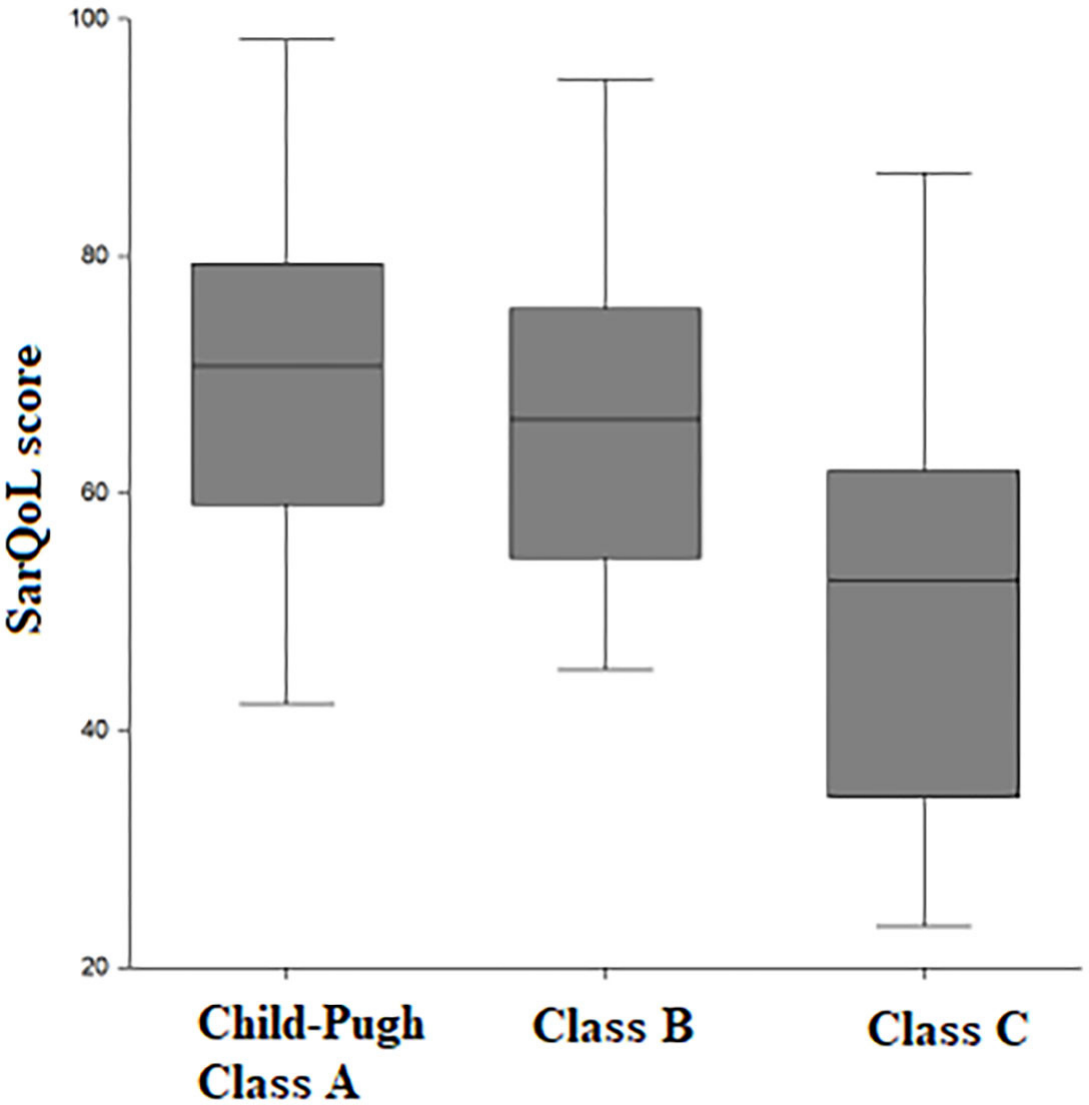
Distribution of SarQoL score according to Child-Pugh score.

A total of 71 patients with liver cirrhosis were included in the study (Table 1); 53.5% of the included subjects fulfilled the criteria for sarcopenia according to the EWGSOP definition and measurement of skeletal muscle index (SMI) at CT scan. There was a statistically significant higher proportion of patients with sarcopenia as liver function worsened. Specifically, sarcopenia was present in 31.2% of patients with Child-Pugh class A, in 58.3% of patients with class B, and in 93.5% with class C (*p* = 0.0003). The SarQoL® score was statistically significantly lower in Child-Pugh class C vs. class B and class A (70.2 vs. 66.5 vs. 52.5 points, *p* = 0.0002) (Figure 1).

**Table 1:** Characteristics of the whole analysed cohort.

**Characteristics**	**Population (*N* = 71)**
Age (years)	
Mean	54.5 ± 12.6
Median	54 (22.3–83)
Gender	
Male	48 (67.6)
Female	23 (32.4)
Aetiology of liver cirrhosis	Alcohol 32.4% Hepatitis c 26.8% Hepatitis b 7.1% Hepatitis b and d 25.3% Other 8.4% (autoimmune, cholestatic diseases, Wilson disease)
Child-Pugh classification	45.1% class A 33.8% class B 21.1% class C
Mean MELD-Na score	14.9 ± 6.1
Mean BMI (body mass index) kg/m^2^	26.4 ± 4.2
Mean SarQoL® score	64.9 ± 16.9

Patients were analysed according to the presence or absence of sarcopenia as is detailed in Table 2. Patients with liver cirrhosis and sarcopenia were statistically and significantly sicker compared to patients without sarcopenia, particularly, with higher MELD-Na and Child-Pugh scores, higher rate of infections, large/refractory ascites, and more episodes of hepatic encephalopathy, as well as lower values of platelets.

**Table 2:** Summary of patients' characteristics according to presence or absence of sarcopenia.

**Variable**	**Sarcopenia Yes (*n* = 38)**	**Sarcopenia No (*n* = 33)**	***P* value**
**SarQoL^®^ score**	56.3 ± 14.7	74.9 ± 13.5	**<0.0001**
**Age (years)**	57.7 ± 11.8	50.9 ± 12.5	**0.02**
Weight (kg)	78.7 ± 15.9	77.8 ± 13.4	0.87
Body mass index (BMI) (kg/m^2^)	26.2 ± 4.4	26.5 ± 3.9	0.75
**Haemoglobin (g/dL)**	10.9 ± 2.6	12.7 ± 2.1	**0.008**
**MELD-Na score**	17.4 ± 6.5	11.9 ± 3.7	**0.00008**
**Child-Pugh score**	8.6 ± 2.5	6.2 ± 1.3	**0.00001**
**Haemoglobin (g/dL)**	10.9 ± 2.6	12.7 ± 2.1	**0.008**
**Albumin (g/dL)**	3.2 ± 0.6	3.9 ± 0.6	**0.00002**
**Total bilirubin (mg/dL)**	3.2 ± 2.4	1.7 ± 1.2	**0.004**
**Sodium (mmol/L)**	134.9 ± 7.1	139 ± 3.2	**0.006**
**Platelet count/mm** ^**3**^	91.9 ± 47.5	127.4 ± 81.3	**0.04**
Lymphocytes/mm^3^	1.07 ± 0.55	1.27 ± 0.54	0.08
**LFI** **<** **3.2**	39.47%	93.94%	**<0.0001**
Hepatocellular carcinoma	36.84%	18.18%	0.08
Spontaneous bacterial peritonitis (SBP)	8.82%	3.13%	0.33
Acute kidney injury	6.06%	3.13%	0.57
**Other infections (except SBP)**	31.43%	3.13%	**0.002**
Portal vein thrombosis	16.67%	18.75%	0.82
**Ascites-large/** **refractory**	50%	12.12%	**0.0006**
**Hepatic encephalopathy episodes**	55.26%	6.06%	**0.00001**
Presence of clinically significant portal hypertension	21.62%	15.15%	0.48
Presence of diabetes mellitus	21.62%	9.38%	0.16
Included on the waiting list for liver transplantation	63.16%	45.45%	0.13
Time elapsed since diagnosis of cirrhosis (months)	83.5 + 67.2	83.9 + 67.2	0.78

*Bold values are the variables that proved to be statistically significant different*.

**Figure 2:**
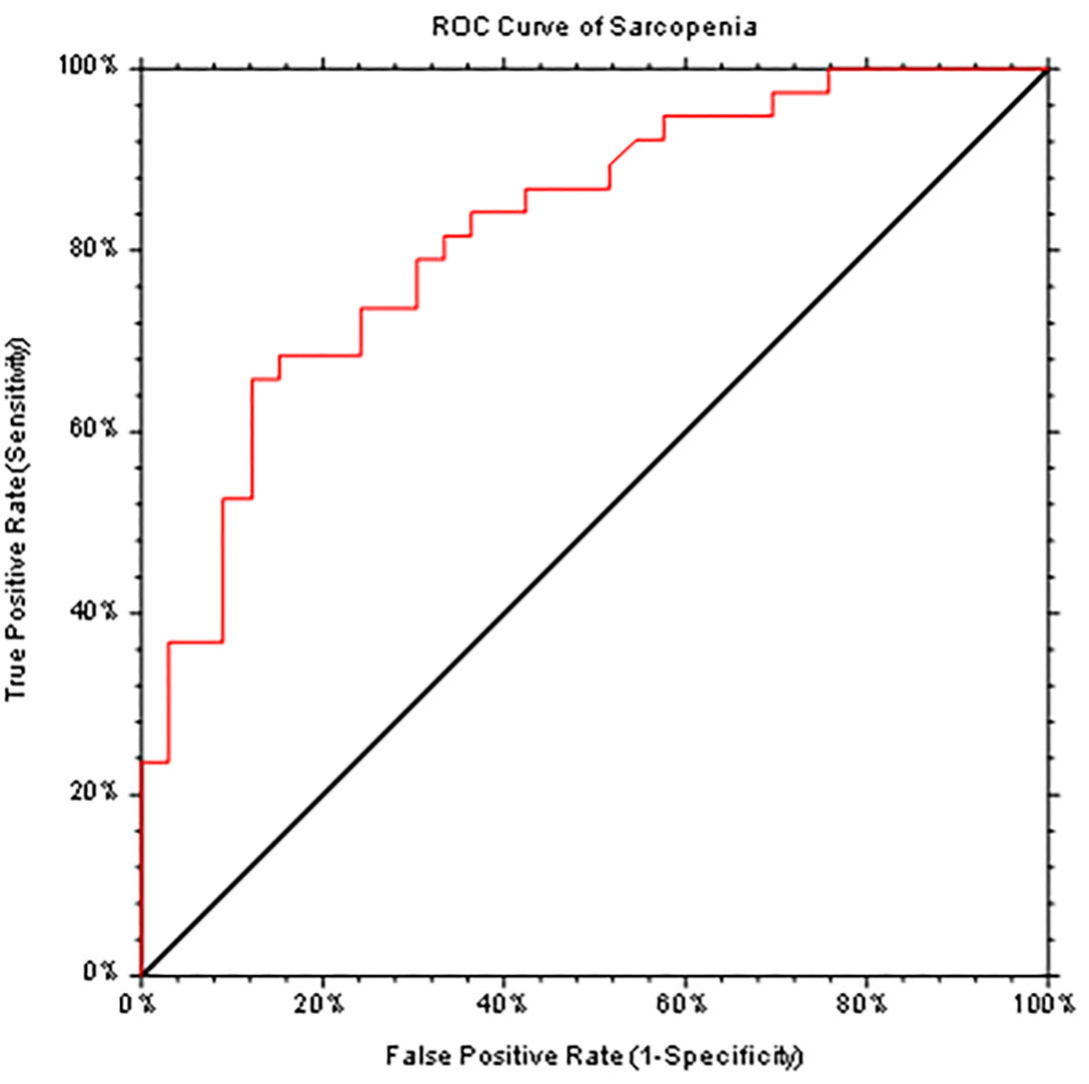
ROC curve (AUC = 0.823) generated by SarQoL® score which accurately diagnoses sarcopenia in patients with end-stage liver diseases.

The SarQoL® score was evaluated according to different complications of cirrhosis, with statistically significant lower scores in patients with sarcopenia, in patients with ascites requiring paracentesis, and in patients with hepatic encephalopathy. The results are presented in Table 3. Even after we excluded patients with Child-Pugh C from the analysis, SarQoL® score was significantly lower in patients with sarcopenia (59.38 ± 10.95) vs. patients without sarcopenia and compensated or with early decompensation (75.58 ± 13.16 points, *p* < 0.0001). There was a statistically significant lower SarQoL® score in patients with LFI >3.2 (49.93 ± 13.44) compared to patients with LFI <3.2 (73.13 ± 12.45, *p* < 0.0001). The AUROC curve was calculated for establishing the statistical performance of SarQoL® score for the diagnosis of sarcopenia (Figure 2). We found that a cut-off level of 75.9 points for SarQoL® score can accurately detect sarcopenia in patients with end-stage liver disease (AUROC of 0.823, SE of 92.1%, and SP of 45.5%, PPV and NPV of 66 and 83.3%, respectively, correctly classified 73.2% of cirrhotic patients with sarcopenia). After excluding patients with Child-Pugh class C cirrhosis, the cut-off value was ≤ 73.5 points for SarQoL® score with an AUROC of 0.827 with SE of 90.1%, SP of 56.1%, and PPV and NPV of 60.7 and 98.1%, respectively, correctly classified 78% of cirrhotic patients with sarcopenia and Child-Pugh class A and B.

**Table 3:** SarQoL® score distribution according to different complications of cirrhosis.

**Type of complication of cirrhosis**	**SarQoL^®^ score in patients with the specified complication**	**SarQoL^®^ score in patients without the specified complication**	***P* value**
Hepatocellular carcinoma	62.32 ± 15.07	66.66 ± 17.16	0.19
Spontaneous bacterial peritonitis (SBP)	56.06 ± 30.59	66.26 ± 16.16	0.58
Acute kidney injury	63.60 ± 4.39	65.85 ± 17.21	0.77
Other infections (except SBP)	57.62 ± 18.06	67.28 ± 16.13	0.10
Portal vein thrombosis	91.9 ± 47.5	127.4 ± 81.3	0.71
**Ascites-large/refractory**	54.25 ± 14.87	70.53 ± 14.86	**0.0002**
**Hepatic encephalopathy episodes**	55.53 ± 17.23	69.48 ± 14.93	**0.002**
Presence of clinically significant portal hypertension	65.94 ± 19.07	64.99 ± 12.71	0.78

*Bold values are the variables that proved to be statistically significant different*.

## Discussion

In patients with cirrhosis evaluated for liver transplantation, sarcopenia and frailty are increasingly recognised as independent predictors of clinical outcomes including wait-list mortality and reduced survival after surgery. Evaluation of body mass composition and assessment of frailty are becoming increasingly important in the management of liver transplant candidates and recipients and their proactive treatment may improve outcomes (7). Sarcopenia represents an important economic and social burden, and improvements in QoL for people with liver cirrhosis and sarcopenia should be a priority for future interventions designed to prevent or treat sarcopenia (16). A positive frailty or sarcopenia screen should prompt evaluation for underlying etiologic risk factors and the development of an ambulatory personalised management plan as stated in the recent AASLD guideline (2).

The symptom prevalence of patients with end-stage liver disease resembles that of patients with other advanced conditions. The most frequently reported symptoms were pain, breathlessness, muscle cramps, sleep disturbance, depression, anxiety, and erectile dysfunction. Decompensation led to a significant worsening of health-related QoL as was previously demonstrated (9). In our study, patients with sarcopenia had significantly advanced liver failure and a significantly higher rate of important portal hypertension, but also a significantly lower QoL quantified by the SarQoL® score. The pathogenesis of sarcopenia is multifactorial and favouring factors are interrelated. There are several studies (5, 17–19) demonstrating that incorporating sarcopenia in the conventional prognostic factors had added value, particularly in compensated and early decompensated cirrhosis. This was confirmed by our study regarding the screening of patients with Child-Pugh A and B cirrhosis by SarQoL® questionnaire. Subclassification of prognostic factors according to sarcopenia may help to better assess the prognosis of cirrhosis and intervene through different strategies (exercise, nutrition, and psychological support).

Screening for sarcopenia by a simple easy-to-do questionnaire that reflects low QoL related to sarcopenia was never done. This is the first study that validated this SarQoL® score in end-stage liver disease and also proved to be a valuable tool for screening of patients with cirrhosis for sarcopenia (a good clinical value with an AUROC of >0.8, a SE >90%, and an NPV >90%). Gasparik et al. (20) already proved that the Romanian version of the SarQoL® questionnaire is conceptually and literally equivalent with the source instrument, qualified in terms of psychometric properties, and can be a useful tool for assessing a sarcopenia-related QoL among frail elderly individuals. A recent study (21) demonstrated that the SarQoL® questionnaire can discriminate between robust, pre-frail, and frail subjects with declining QoL scores according to the category of frailty. There was also a significant difference in our cohort between robust (LFI <3.2) and prefrail/frail (LFI > 3.2) patients regarding the obtained SarQoL® score. In other words, the SarQoL® questionnaire is able to discriminate on more than just the physical aspects of QoL. Particularly, it brings extra precision in being able to discriminate between robust, frail, and pre-frail individuals. Despite this, it needs further investigation in this regard in patients with liver cirrhosis awaiting liver transplantation.

The strength of the study is represented by the first time use of SarQoL® score in liver cirrhosis and its correlation with advanced liver failure and complications of cirrhosis (mainly due to portal hypertension).

Our study revealed that, in patients with liver cirrhosis, even in those with low Child-Pugh and low MELD scores, QoL can be severely affected by the presence of sarcopenia. In addition, its identification and reversal are required by a combination of nutritional, physical, pharmacological, and psychological interventions in order to improve the prognosis of patients with end-stage liver disease. The SarQoL® questionnaire is an already validated tool for assessing sarcopenic individuals. It can easily and repeatedly be applied to prevent and assess response to therapy of sarcopenia in specific populations, especially in patients with liver cirrhosis who are of high priority for LT or in patients avoiding futile LT. One limitation of our study is the relatively small sample size; we intend to validate our cut-off for the SarQoL® score on another cohort evaluated in our Hepatology Department.

Hence, the SarQoL® questionnaire could establish the patients that would benefit mostly following a multidisciplinary approach and therapeutic interventions.

## Data Availability Statement

The original contributions presented in the study are included in the article/supplementary material, further inquiries can be directed to the corresponding author/s.

## Ethics Statement

The studies involving human participants were reviewed and approved by Local Hospital Committee. Written informed consent for participation was not required for this study in accordance with the national legislation and the institutional requirements. Written informed consent for all performed procedures was signed by patients at admittance into the hospital.

## Author Contributions

SI: conceptualization and Writing-original draft. SI and RV: methodology. SI and RI: statistical analysis. SI, VM, MM, VB, DB, and GI: data collection. SI, RI, CG, and LG: writing, reviewing, and editing. LG: visualization. All authors contributed to the article and approved the submitted version.

## Conflict of Interest

The authors declare that the research was conducted in the absence of any commercial or financial relationships that could be construed as a potential conflict of interest.

## Publisher's Note

All claims expressed in this article are solely those of the authors and do not necessarily represent those of their affiliated organizations, or those of the publisher, the editors and the reviewers. Any product that may be evaluated in this article, or claim that may be made by its manufacturer, is not guaranteed or endorsed by the publisher.
